# The 12-item Self-Report World Health Organization Disability Assessment Schedule (WHODAS) 2.0 Administered Via the Internet to Individuals With Anxiety and Stress Disorders: A Psychometric Investigation Based on Data From Two Clinical Trials

**DOI:** 10.2196/mental.7497

**Published:** 2017-12-08

**Authors:** Erland Axelsson, Elin Lindsäter, Brjánn Ljótsson, Erik Andersson, Erik Hedman-Lagerlöf

**Affiliations:** ^1^ Division of Psychology Department of Clinical Neuroscience Karolinska Institutet Stockholm Sweden; ^2^ Gustavsberg Primary Care Clinic Gustavsberg Sweden; ^3^ Centre for Psychiatry Research Department of Clinical Neuroscience Karolinska Institutet Stockholm Sweden; ^4^ Osher Center for Integrative Medicine Department of Clinical Neuroscience Karolinska Institutet Stockholm Sweden

**Keywords:** disability, Internet, psychometrics, questionnaire, validity, WHODAS

## Abstract

**Background:**

The World Health Organization Disability Assessment Schedule 2.0 (WHODAS 2.0) is a widespread measure of disability and functional impairment, which is bundled with the Diagnostic and Statistical Manual of Mental Disorders (Fifth Edition) for use in psychiatry. Administering psychometric scales via the Internet is an effective way to reach respondents and allow for convenient handling of data.

**Objective:**

The aim was to study the psychometric properties of the 12-item self-report WHODAS 2.0 when administered online to individuals with anxiety and stress disorders. The WHODAS 2.0 was hypothesized to exhibit high internal consistency and be unidimensional. We also expected the WHODAS 2.0 to show high 2-week test-retest reliability, convergent validity (correlations approximately .50 to .90 with other self-report measures of functional impairment), that it would differentiate between patients with and without exhaustion disorder, and that it would respond to change in primary symptom domain.

**Methods:**

We administered the 12-item self-report WHODAS 2.0 online to patients with anxiety and stress disorders (N=160) enrolled in clinical trials of cognitive behavior therapy, and analyzed psychometric properties within a classical test theory framework. Scores were compared with well-established symptom and disability measures, and sensitivity to change was studied from pretreatment to posttreatment assessment.

**Results:**

The 12-item self-report WHODAS 2.0 showed high internal consistency (Cronbach alpha=.83-.92), high 2-week test-retest reliability (intraclass correlation coefficient=.83), adequate construct validity, and was sensitive to change. We found preliminary evidence for a three-factorial structure, but one strong factor accounted for a clear majority of the variance.

**Conclusions:**

We conclude that the 12-item self-report WHODAS 2.0 is a psychometrically sound instrument when administered online to individuals with anxiety and stress disorders, but that it is probably fruitful to also report the three subfactors to facilitate comparisons between studies.

**Trial Registration:**

Clinicaltrials.gov NCT02540317; https://clinicaltrials.gov/ct2/show/NCT02540317 (Archived by WebCite at http://www.webcitation.org/6vQEdYAem); Clinicaltrials.gov NCT02314065; https://clinicaltrials.gov/ct2/show/NCT02314065 (Archived by WebCite at http://www.webcitation.org/6vQEjlUU8)

## Introduction

The World Health Organization Disability Assessment Schedule 2.0 (WHODAS 2.0) [1,2] is an assessment tool developed by the World Health Organization (WHO) to measure disability and functional impairment in accordance with the International Classification of Functioning, Disability and Health [3]. The WHODAS 2.0 comes bundled with the *Diagnostic and Statistical Manual of Mental Disorders* (Fifth Edition; *DSM-5*), and is endorsed as a new and useful measure of functional impairment in psychiatric disorders [4]. The WHODAS 2.0 measures average functioning in everyday situations for the last 30 days, and surveys six domains of functioning: (1) cognition (understanding and communicating), (2) mobility (ability to move and get around), (3) self-care (eg, with regard to hygiene, dressing, and eating) (4) getting along with others, (5) life activities (ability to attend to everyday responsibilities), and (6) participation in society [1]. The most widespread and evaluated form of the WHODAS 2.0 is the 36-item structured interview version, which takes approximately 20 minutes to complete and has excellent psychometric properties [2]. This study, however, concerns the shorter 12-item self-assessment questionnaire version of the WHODAS 2.0. In the WHODAS 2.0 field trials, the reduced 12-item scale, despite only taking approximately 5 minutes to complete, explained 81% of the variance in the 36-item scale [2]. Several large-scale studies have suggested that the 12-item WHODAS 2.0 is a reliable and valid instrument when administered as an interview [5,6] or in a pencil-and-paper format [7,8]. As to dimensionality, both a one-factor structure [6,8] and a second-order model that specifies the six WHODAS 2.0 domains of functioning as subfactors to an overarching disability variable [5,6] have been suggested.

Administering psychometric questionnaires via the Internet is rapidly becoming more common in both research and routine mental care. Compared with conventional pencil-and-paper administration, there are many advantages of this online approach. Respondents can complete the necessary questionnaires wherever an Internet connection is available, and for the clinician or researcher data are quickly and easily stored, scored, analyzed, and interpreted with less risk for human error. Questionnaires are easily integrated with routine care software for evaluation and record keeping, as well as digital monitoring systems and Web-based psychological treatments. Loss of individual item scores may be prevented entirely, time of measurement may be registered and determined by prespecified time schedules, and respondents may be readily contacted via automatic email or text-message reminders. Although it has often been found that well-established scales do well regardless of administration format, online adaptions of validated scales should preferably undergo separate validation [9,10]. There is thus a need for separate validation of the self-rated 12-item WHODAS 2.0 when administered via the Internet and, to the knowledge of the authors, no study has yet investigated the convergent or discriminant validity, responsiveness, test-retest reliability, or factor structure of the self-rated 12-item WHODAS 2.0 when administered online to individuals with common mental disorders.

Based on data from two clinical trials of cognitive behavior therapy (CBT) for anxiety and stress disorders, we aimed to present estimates of test-retest reliability and thoroughly investigate item score distributions, convergent and discriminant validity, as well as the factor structure of the 12-item online WHODAS 2.0 when administered to individuals with anxiety and stress disorders. We expected the scale to be unidimensional, possibly with the six domains of functioning as subfactors (see previous), and with high internal consistency (Cronbach alpha>.80) as seen in previous studies. We expected strong baseline Pearson correlations (approximately .50 to .90) between the WHODAS 2.0 and other measure of disability or functional impairment, as well as substantial, yet slightly weaker, baseline Pearson correlations (approximately .30 to .70) with the measures of depression and general anxiety. We expected the WHODAS 2.0 to discriminate well between chronic stress patients with and without *International Classification of Diseases, Tenth Revision* (*ICD-10*) exhaustion disorder, a disorder characterized by burnout-like symptoms including fatigue and cognitive weariness, and typically regarded as highly disabling [11]. We also hypothesized that the WHODAS 2.0 would be sensitive to within-group change in primary psychiatric symptom domain.

## Methods

### Design

This was a psychometric study of the WHODAS 2.0 administered online to patients with anxiety and stress disorders. Data were collected from clinical trials of CBT for severe health anxiety (n=60) and stress disorders (n=100) conducted at Karolinska Institutet and Gustavsberg primary care clinic, Stockholm, Sweden. Both trials were approved by the Stockholm regional ethics review board (2015/415-31/5, 2014/1530-31/2), registered at ClinicalTrials.gov (NCT02540317, NCT02314065), and participants provided informed consent. Data used for this study were collected between September 2015 and July 2016.

### Recruitment

Both clinical trials employed patient self-referral via the Internet, and advertised in newspapers as well as on online social media networks. Study applicants completed a series of online screening symptom questionnaires before a diagnostic interview with a licensed psychologist. This interview primarily served to survey eligibility criteria and lead up to a decision regarding inclusion or exclusion (ie, this decision was based on the psychiatric interview), but also served to collect important clinical data (eg, comorbid diagnoses). After the pretreatment assessment, which was conducted online, patients underwent randomization and subsequent treatment. All included patients were at least 18 years of age. The severe health anxiety sample had a principal diagnosis of *DSM-5* somatic symptom disorder or illness anxiety disorder, whereas the stress disorders sample had a principal diagnosis of *DSM-5* adjustment disorder or *ICD-10* exhaustion disorder (for a brief introduction to this disorder, see [11]). We intend to provide an in-depth description of the methods of the clinical trials, including the recruitment process, in the primary publications.

### Procedure

All questionnaires were completed through a simple Web-based interface with white background, radio buttons, and checkboxes. All 60 patients in the severe health anxiety sample and 50 patients in the stress disorders trial received CBT (12 weeks, disorder-specific) for their principal disorder. The WHODAS 2.0 was administered before and after CBT. In addition, patients with severe health anxiety completed the WHODAS 2.0 at screening, thus allowing for estimates of test-retest reliability. Other measures used to validate the WHODAS 2.0 were also administered before and after treatment.

### Clinical Instruments for Diagnostic Assessment

Both *DSM-5* somatic symptom disorder and illness anxiety disorder were assessed with the Health Preoccupation Diagnostic Interview, which exhibits excellent interrater reliability [12]. Both *ICD-10* exhaustion disorder and *DSM-5* adjustment disorder were assessed with a clinical interview developed specifically for the stress disorders trial that closely followed the diagnostic criteria of *ICD-10* and *DSM-5*. Comorbid psychiatric disorders were surveyed with the Mini-International Neuropsychiatric Interview, which is a reliable and valid instrument for assessing psychiatric disorders [13].

### Self-Rated Measures of Functional Impairment

The self-report 12-item WHODAS 2.0 [2] instructs the respondent to determine his or her difficulty in engaging in particular activities (eg, “taking care of [...] household responsibilities” and “maintaining a friendship”), as rated on a scale from “none” (no difficulty) to “extreme or cannot do” and corresponding to six domains of functioning (see Introduction). We employed the WHO simple scoring method [1] that gives a 12-item WHODAS 2.0 score range from 12 to 60, where higher scores indicate higher disability or loss of function. This type of straightforward additive scoring has been shown to correlate strongly (*r*>.98, ρ=.999) with more complex scoring methods incorporating weights based on item response patterns [5,14]. Just like the pencil-and-paper version, the WHODAS 2.0 was presented over two pages with items 1 to 5 on the first page, and items 6 to 12 on the second page.

The Sheehan Disability Scale (SDS) is a well-established three-item measure of psychiatric symptom-related functional impairment with a sum score range from 0 to 30, with a higher score indicating a higher degree of functional impairment [15,16]. The Work Ability Index (WAI) measures work ability with a sum score range from 7 to 49, and higher score indicating higher work ability [17-19]. The SDS and WAI were used as indexes of functional impairment.

### Self-Rated Measures of Primary Psychiatric Symptoms

The Health Anxiety Inventory (HAI) is a 64-item questionnaire that measures health anxiety on a scale from 0 to 192, with a higher score indicating more health anxiety [20-22]. The Perceived Stress Scale (PSS) is a 14-item measure of perceived stress with a range from 0 to 56, with a higher score indicating more stress [23]. The HAI and PSS were used to describe the samples in terms of primary symptom domains (ie, health anxiety and stress).

### Self-Rated Measures of General Psychiatric Symptoms

The self-reported Montgomery-Åsberg Depression Rating Scale (MADRS-S) is a widely used nine-item questionnaire that measures depressive symptoms on a scale from 0 to 54, with higher scores indicating more symptoms of depression [24-26]. The seven-item Generalized Anxiety Disorder scale (GAD-7) measures general anxiety from 0 to 21, with higher scores indicating more general anxiety [27]. The MADRS-S and GAD-7 were used to assess the common symptom domains of depression and general anxiety and facilitate comparison of the two samples.

### Statistical Analysis

Analyses were done in SPSS version 23.0.0.2 (IBM Corp, Armonk, NY, USA) and R 3.3.2 [28] with lavaan 0.5-22 [29]. We pooled the severe health anxiety and stress disorders samples (total N=160), dropped three multivariate outliers, and employed structural equation modeling to assess the validity of the simple one-factor model of disability endorsed by the WHO [1], as well as the second-order model fitted in two previous studies [5,6]. The latter had latent variables corresponding both to the six WHODAS 2.0 dimensions of functioning and an overarching latent disability variable. We employed weighted least squares means and variance adjusted estimation, which is adequate for categorical data and nonnormal manifest variables [30]. Based on the recommendations of Byrne [31], we established a priori criteria for acceptable model fit in terms of a comparative fit index (CFI) greater than 0.90, a Tucker-Lewis index (TLI) greater than 0.90, and a root mean square error of approximation (RMSEA) of 0.08 or lower. Post hoc exploratory factor analysis was based on principal axis factoring with promax rotation (ie, factors were assumed to be correlated).

Internal consistency was investigated in terms of Cronbach alpha, complemented by adjusted item-total correlations (ITCs), which are not as strongly affected by the number of scale items. For instruments of typical length, Cronbach alpha≥.9 is usually regarded as excellent, ≥8 as good, and ≥7 as acceptable. Test-retest reliability was estimated based on a two-way mixed-effects model absolute agreement intraclass correlation coefficient (ICC) and data from a subsample (n=25) from the severe health anxiety trial that had completed the screening and pretreatment assessments within 14 days (mean 6.8, SD 3.2, range 1-13).

We used an independent samples *t* test to assess if patients with and without a clinical diagnosis of exhaustion disorder differed with regard to WHODAS 2.0 score, and then performed a receiver operating characteristic analysis to assess the ability (area under the curve [AUC]) of the WHODAS 2.0 to identify cases of exhaustion disorder. Pearson correlations were used to investigate baseline associations between the WHODAS 2.0 and other self-rated measures.

To evaluate sensitivity to change, we compared pretreatment and posttreatment mean scores using paired samples *t* test and calculated effect sizes as the difference in means at t_1_ and t_2_, divided by the t_1_ standard deviation. Based on the Jacobson and Truax reliable change index [32], we classified patients as either reliably improved (reduction in the HAI>29.9 or reduction in the PSS>7.1) or not reliably improved. As recommended in the assessment of responsiveness, we also further differentiated between reliably improved patients based on whether they improved more than a minimal important difference in their primary symptom domain [33]. In other words, for both samples we estimated change in functional impairment in three strata: (1) patients who did not made a reliable improvement on the HAI or PSS, (2) patients who reliably improved but only to a slight to moderate degree, and (3) patients who reliably improved to a large degree. However, it was necessary to set the threshold for changing more than a minimal important difference relatively high (1.5 SD) due to the precision of the instruments requiring a relatively large score change (1.21 SD in the case of HAI, 1 SD in the case of PSS) for patients to have experienced true change with 95% certainty, as assessed with the reliable change index [32]. Item-level change was assessed with the Wilcoxon signed-rank test.

The WHODAS 2.0 response rate was 100% for the screening and pretreatment assessments. Because there was a small proportion of missing posttreatment data (for the WHODAS 2.0: 3%, 3/100 in the stress sample; 5%, 3/60 in the severe health anxiety sample), and we did not aim to investigate treatment effects but rather the responsiveness of the scale, data were used on a complete case (not intention-to-treat) basis.

## Results

### Sample Characteristics

Sample characteristics for the severe health anxiety sample, the stress disorders sample, and the pooled sample are presented in Table 1.

### Factor Structure

Neither the one-factor model endorsed by the WHO (χ^2^_54_=1699.1, *P<*.001; CFI=0.29, TLI=0.14, RMSEA=0.44, standardized root mean square residual [SRMR]=0.52) nor the second-order model presented by previous studies achieved acceptable fit (χ^2^_48_=132.7, *P<*.001; CFI=0.96, TLI=0.95, RMSEA=0.11, SRMR=0.09), and we did not see implementing any of the modification indexes as theoretically justifiable.

**Table 1:** Sample characteristics.

Measure		Severe health anxiety (n=60)	Stress disorders (n=100)	Total (N=160)
**Demographics**				
	Age (years), mean (SD^a^), range	36.4 (11.9), 18-78	46.2 (8.8), 26-65	42.5 (11.1), 18-78
	Gender (female), n (%)	40 (67)	85 (85)	125 (78)
**Psychiatric symptoms^b^**				
	HAI, mean (SD), range	105.6 (24.7), 51-164	—	—
	PSS, mean (SD), range	—	36.8 (7.1), 17-52	—
	MADRS-S, mean (SD), range	13.9 (7.4), 1-34	19.7 (7.5), 3-40	17.5 (7.9), 1-40	
	GAD-7, mean (SD), range	12.0 (5.3), 2-21^c^	10.8 (4.8), 2-21	11.2 (5.0), 2-21^c^
	MDD, n (%)	11 (18)	13 (13)	24 (15)
	≥1 anxiety disorder/OCD^d^, n (%)	34 (57)	19 (19)	53 (33)
**Functional impairment^e^**				
	WHODAS 2.0, mean (SD), range	21.1 (6.3), 12-36	24.7 (8.5), 12-51	23.4 (7.9), 12-51
	SDS, mean (SD), range	10.4 (6.7), 0-26	—	—
	WAI, mean (SD), range	—	33.0 (8.4), 13.0-47.0	—
	On sick leave, n (%)	3 (5)	14 (14)	17 (11)

^a^SD: standard deviation.

^b^HAI: Health Anxiety Inventory; PSS: Perceived Stress Scale; MADRS-S: Montgomery-Åsberg Depression Rating Scale self-report version; GAD-7: Generalized Anxiety Disorder 7-item scale; MDD: major depressive disorder; OCD: obsessive compulsive disorder;.

^c^GAD-7 data only available from a subsample of the severe health anxiety sample (n=43).

^d^At least one anxiety or obsessive compulsive disorder that is not severe health anxiety.

^e^WHODAS 2.0: World Health Organization Disability Assessment Schedule 2.0; SDS: Sheehan Disability Scale; WAI: Work Ability Index.

**Table 2:** Factor loadings of the WHODAS 2.0^a^.

Item	Factor 1: psychosocial^b^	Factor 2: self-care^b^	Factor 3: mobility^b^	A priori dimension^c^
1. Standing long periods	–.008	–.097	.877	Mobility
2. Household responsibilities	.540	–.027	.264	Household
3. Learning new tasks	.740	–.002	.035	Cognitive
4. Joining community activities	.848	–.046	.042	Society
5. Emotionally affected	.652	–.087	.074	Society
6. Concentrating	.683	–.010	–.040	Cognitive
7. Walking long distance	–.003	.329	.597	Mobility
8. Washing whole body	–.068	.893	.132	Self-care
9. Getting dressed	.078	.909	–.126	Self-care
10. Dealing with strangers	.704	.167	–.164	Social
11. Maintaining friendships	.618	.128	–.101	Social
12. Work/school activities	.814	–.059	.037	Household

^a^WHODAS 2.0: World Health Organization Disability Assessment Schedule 2.0.

^b^Factor loadings (regression coefficients) based on principal axis factoring with promax rotation.

^c^Cognitive: understanding and communicating; household: life activities; mobility: getting around; social: getting along with others; society: participation in society.

Post hoc exploratory factor analysis (Kaiser-Meyer-Olkin test=0.85, Barlett test<.001) was suggestive of a three-factor solution, with one very strong factor (eigenvalue=5.4, 45.0% of variance explained) and two weak factors (eigenvalues=1.1-1.7, 9.3%-14.2% of variance explained).

After rotation (Table 2) it was clear that one of the two weak factors was primarily associated with items 1 and 7 (“getting around”), and the other weak factor was primarily associated with items 8 and 9 (“self-care”), whereas the strong factor was associated with the remaining items (“understanding and communicating,” “getting along with others,” “life activities,” and “participation in society”). Interfactor correlations were substantial (*r*=.40-.46). Dropping items 1, 7, 8, and 9 had little impact on interindividual sum score variance, and the resulting mean was heavily correlated with the conventional one (*r=*.97). Thus, the factor analysis indicated that a majority of items had high loadings on a general disability factor, but that “mobility” and “self-care” emerged as distinct, but weak, factors.

### Item Score Distributions

Parameters related to item score distributions are presented in Table 3. Overall, patients scored low on items that concerned functional impairment in self-care, and higher on items that concerned difficulties participating in society and everyday life activities, as well as items that concerned cognitive impairment. Unlike the summary score, several item distributions were skewed and showed high kurtosis. There was also evidence of a floor effect with regard to several items.

### Internal Consistency

Adjusted baseline ITCs are presented in Table 3, where the mean adjusted ITC was 0.60 (SD 0.11). Cronbach alpha values were good to excellent for both the total sum score and the proposed psychosocial subscale (Table 4).

### Test-Retest Reliability

The test-retest reliability of the WHODAS 2.0, based on the severe health anxiety sample data (n=25), was estimated at ICC=0.83 (95% CI 0.62-0.92) and the correlation between the measurements was *r=*.71. The test-retest reliability of the proposed psychosocial subscale was similar (ICC=0.85, 95% CI 0.67-0.94; *r*=.75).

### Construct Validity: Associations With Other Measures

In the stress disorders sample, patients with a principal diagnosis of exhaustion disorder had a significantly higher WHODAS 2.0 mean score than those who had a principal diagnosis of adjustment disorder (mean difference 8.84, 95% CI 5.96-11.72; *t*_98_=6.10, *P*<.001), and the WHODAS 2.0 discriminated well between stress patients with and without exhaustion disorder (AUC=0.81, 95% CI 0.73-0.89; *P*<.001). The proposed psychosocial subscale fared about as well (AUC=0.79, 95% CI 0.70-0.87; *P*<.001). Baseline correlations between the WHODAS 2.0 as well as the subscale and other self-assessment questionnaires are shown in Table 5.

### Sensitivity to Change

Effect sizes and tests pertaining to responsiveness are presented in Table 6. Wilcoxon signed-rank tests showed that all but one WHODAS 2.0 item (item 9, related to self-care) changed in the severe health anxiety sample, whereas 9 of 12 items changed (but not items 1, 8, or 9—one mobility and both self-care items) in the stress disorders sample.

**Table 3:** Web-based self-report 12-item WHODAS 2.0 item scores^a^.

Item and subscale		Mean (SD^b^)	Median (range)	Floor	Ceiling	Skew	Kurtosis	ITC^c^
**Item**								
	1. Standing long periods	1.54 (0.89)	1 (1-4)	66%	0%	1.58	1.47	0.44
	2. Household responsibilities	2.07 (1.02)	2 (1-4)	38%	0%	0.47	–1.00	0.64
	3. Learning new tasks	1.87 (1.02)	2 (1-5)	49%	1%	0.85	–0.37	0.70
	4. Joining community activities	2.23 (1.19)	2 (1-5)	38%	4%	0.55	–0.77	0.78
	5. Emotionally affected	3.21 (1.14)	4 (1-5)	13%	6%	–0.69	–0.60	0.59
	6. Concentrating	2.37 (1.05)	2 (1-5)	26%	1%	0.17	–0.90	0.61
	7. Walking long distance	1.51 (0.82)	1 (1-5)	66%	1%	1.63	2.29	0.52
	8. Washing whole body	1.20 (0.55)	1 (1-4)	86%	0%	3.12	10.22	0.48
	9. Getting dressed	1.16 (0.47)	1 (1-4)	88%	0%	3.44	12.87	0.48
	10. Dealing with strangers	1.83 (1.01)	2 (1-5)	49%	1%	1.10	0.43	0.66
	11. Maintaining friendships	1.85 (1.07)	1 (1-5)	53%	1%	0.97	–0.17	0.59
	12. Work/school activities	2.54 (1.20)	3 (1-5)	25%	6%	0.27	–0.88	0.75
	Total score	23.37 (7.91)	23 (12-51)	8%	0%	0.68	0.52	—
**Subscale**								
	Psychosocial	12.60 (4.70)	12.76 (5.60-24.84)	8%	0%	0.31	–0.49	—
	Self-care	2.12 (0.86)	1.80 (1.80-7.21)	84%	0%	3.20	11.23	—
	Mobility	2.25 (1.15)	1.47 (1.47-6.49)	56%	0%	1.56	1.76	—

^a^WHODAS 2.0: World Health Organization Disability Assessment Schedule 2.0. Based on baseline data from two clinical trials of severe health anxiety and stress disorders (total N=160, items scored from 1 to 5).

^b^SD: standard deviation.

^c^ITC: adjusted item-total correlation.

**Table 4:** Web-based self-report 12-item WHODAS 2.0^a^ internal consistency.

Sample		Screening		Pretreatment		Posttreatment	
		Cronbach alpha	n	Cronbach alpha	n	Cronbach alpha	n
**Total score**							
	Severe health anxiety	.83	60	.86	60	.87	56
	Stress disorder	—	—	.90	100	.92	97
**Psychosocial subscale^b^**							
	Severe health anxiety	.82	60	.88	60	.87	56
	Stress disorder	—	—	.89	100	.91	97

^a^WHODAS 2.0: World Health Organization Disability Assessment Schedule 2.0.

^b^Psychosocial subscale with regression weights applied.

**Table 5:** WHODAS 2.0 associations (bivariate Pearson correlations) with other self-rated questionnaires^a^.

	WHODAS 2.0^b^	Psychosocial subscale	MADRS-S^b^	GAD-7^b^	SDS^b^
WHODAS 2.0	—	.96^c^	.60^c^	.58^c,d^	.66^c^
Psychosocial subscale	.97^e^	—	.65^c^	.54^c,d^	.67^c^
MADRS-S	.65^e^	.64^e^	—	.54^c,d^	.59^c^	
GAD-7	.45^e^	.45^e^	.71^e^	—	.40^c^
WAI	–.71^e^	–.71^e^	–.55^e^	–.28^e^	—

^a^All bivariate Pearson correlations significant at α=.05.

^b^WHODAS 2.0: World Health Organization Disability Assessment Schedule 2.0; MADRS-S: Montgomery-Åsberg Depression Rating Scale-self-report version; GAD-7: Generalized Anxiety Disorder 7-item scale; SDS: Sheehan Disability Scale; WAI: Work Ability Index.

^c^Severe health anxiety sample (n=60).

^d^GAD-7 data only available from a subsample of the severe health anxiety sample (n=43).

^e^Stress disorder sample (n=100).

**Table 6:** Responsiveness stratified by change in primary symptom domain^a^.

Change in primary symptom domain^b^			n (%)	ES^c^	Mean change (95% CI)	*P*^d^
**Severe health anxiety sample**						
	**HAI reliably improved >1.5 SD^e^**		32 (5)			
		WHODAS 2.0		1.21	7.19 (5.17, 9.22)	<.001
		SDS		1.00	7.28 (4.59, 9.98)	<.001
	**HAI reliably improved ≤1.5 SD**		8 (14)			
		WHODAS 2.0		0.52	2.75 (–0.85, 6.35)	.11
		SDS		0.53	1.88 (–1.07, 4.82)	.18
	**HAI not reliably improved**		18 (31)			
		WHODAS 2.0		0.29	2.00 (–0.13, 4.13)	.06
		SDS		0.22	1.39 (–0.59, 3.37)	.16
**Stress disorder sample**						
	**PSS reliably improved >1.5 SD**		33 (34)			
		WHODAS 2.0		0.80	6.42 (4.27, 8.58)	<.001
		WAI		–0.49	–4.14 (–5.75, –2.52)	<.001
	**PSS reliably improved ≤1.5 SD**		12 (12)			
		WHODAS 2.0		0.39	2.83 (0.09, 5.58)	.04
		WAI		–0.25	–2.29 (–5.10, 0.51)	.10
	**PSS not reliably improved**		52 (54)			
		WHODAS 2.0		0.19	1.75 (-0.10, 3.60)	.06
		WAI		–0.13	–0.96 (–2.66, 0.73)	.26

^a^All estimates based on data from patients that completed the posttreatment assessment. Status as improved or not improved based on the Jacobson and Truax reliable change index [32].

^b^HAI: Health Anxiety Inventory; WHODAS 2.0: World Health Organization Disability Assessment Schedule 2.0; SDS: Sheehan Disability Scale; PSS: Perceived Stress Scale; WAI: Work Ability Index.

^c^Standardized effect sizes (ES) are calculated with the stratum-specific pretreatment standard deviation as nominator.

^d^*P* values based on paired samples *t* test.

^e^SD: standard deviation.

## Discussion

### Summary of Principal Results

To our knowledge, this was the first in-depth analysis of the reliability and validity of the self-rated 12-item WHODAS 2.0 when administered online to patients with anxiety and stress disorders. In line with our hypotheses, the WHODAS 2.0 exhibited high internal consistency, acceptable test-retest reliability, and was demonstrated to identify cases of exhaustion disorder. As expected, the WHODAS 2.0 also showed substantial associations with other measures of functioning, as well as slightly weaker but yet substantial associations with primary symptom measures. The instrument was sensitive to change in the primary psychiatric symptom domain, as illustrated by a convincing gradient in change effect size over the nonimproved versus slightly improved versus much improved strata (Table 6). It might be noted that there was no significant change in WHODAS 2.0 for the minimally changed severe health anxiety group, but this is not surprising given the very small size of this subsample (n=8). Taken together, our findings suggest that the self-rated 12-item online WHODAS 2.0—when administered to individuals with anxiety and stress disorders—is a valid measure of disability and functional impairment. The results are important because online administration facilitates handling of data in a wide range of research and routine care settings. Key strengths of this study are that two psychiatric samples with different primary conditions could be examined, that other scales could be used to validate the WHODAS 2.0 scores in both samples, and that the ability of the WHODAS 2.0 to identify patients with a clinical diagnosis of exhaustion disorder could be investigated in the stress disorders sample.

### Factor Structure

Regarding factor structure, we could neither confirm the expected one-factor WHO solution nor the second-order model put forward by previous investigators [5,6]. A post hoc model derived from exploratory analysis saw most items loading heavily (>.5) on one strong factor, and four items (8, 9, 1, and 7) loading heavily on two other factors. The results are somewhat similar to those seen with the WHODAS 2.0 administered to individuals with musculoskeletal pain [34], and suggests that items 8 and 9 (“self-care”), as well as items 1 and 7 (“getting around”), largely tap into sources of variance other than that responsible for most of the sum score (see Table 3). Our subjective impression is that these four items seem more related to physical incapacity than the rest of the scale. Because (1) these items showed apparent evidence of a floor effect (their median sum being zero), (2) the sum score primarily relied on another very strong factor, and (3) the interfactor correlations were moderately strong, the impact of multidimensionality is likely to be very small in the study of change in otherwise healthy samples with anxiety or stress disorders. On the other hand, the interfactorial correlations were small enough to complicate direct comparisons of scores between studies. Especially when anxiety or stress disorder samples differ in terms of somatic comorbidity, it is probably informative to compare the “getting around” and “self-care” subdomains separately. That is, based on the results of this study our tentative recommendation would be for studies on anxiety and stress disorders to report both the self-rated 12-item WHODAS 2.0 sum score, as well as the three subfactors, here referred to as “psychosocial,” “self-care,” and “getting around” (Table 2). However, we wish to emphasize that this factor solution was solely based on an explorative analysis, that the second-order model with six subfactors [5,6] was not far off the mark, and that additional confirmatory factor analyses based on data from similar samples are warranted to arrive at more firm conclusions.

### Score Distribution

On an item level, the most striking aspect of the score distribution was that both items 8 and 9 (ie, the “self-care” items) had a mode score of 1 and appeared to pick up on very little variance in the two samples. This may suggest that individuals with a principal diagnosis of severe health anxiety or a stress disorder are typically not much impaired in terms of ability to carry out everyday self-care tasks such as washing oneself or getting dressed.

### Internal Consistency and Test-Retest Reliability

Although the internal consistency and test-retest reliability estimates were in line with our expectations, and also fulfilled commonly accepted quality criteria for health status questionnaires [35], it is conceivable that the estimates of test-retest reliability were deflated because a psychiatric interview and the decision to include patients in a clinical trial took place between the screening and pretreatment measurements. However, the means from these two measurement points were not significantly different (*t*_59_=1.55, *P*=.13), which for example speaks against patients having altered their responses so as to be included in one of the trials.

### Convergent Validity and Sensitivity to Change

In this study, correlations with other measures of psychiatric symptoms and functional impairment corroborate the construct validity of the self-rated 12-item WHODAS 2.0. As this study was based on data from two clinical trials, we do not find it unfit or surprising that the WHODAS 2.0 was highly correlated with measures of anxiety and depression which were likely to be the primary reasons for functional impairment.

Due to the limited sample size and variability in the size of substrata (nonchanged vs minimally changed vs much changed) we wish to emphasize that the significance tests pertaining to change (Table 6) are of limited value, and emphasize that all *P* values ought to be interpreted alongside their corresponding effect sizes. Over and above the effect size gradient over the nonchanged versus minimally changed versus much changed strata, a general trend was also that the 12-item WHODAS 2.0 changed slightly more from pretreatment to posttreatment than the WAI. This may suggest that the WHODAS 2.0 covers dimensions of functional impairment that, compared to work ability, are more likely to change in a clinical trial of CBT for chronic stress disorders.

### Limitations

The primary limitation of this study is that over and above clinical diagnoses we did not have access to “hard” measures of functional impairment, collected by other means than self-assessment (eg, register data on disability status) that could be used to validate the WHODAS 2.0. One consequence of this is that all indexes of change were to some degree susceptible to social desirability bias or the possibility that patients reported change so as to please their therapist rather than as a consequence of real change in symptoms or disability. However, it has been demonstrated that Web-based survey administration is relatively robust to desirability bias [36], and all scales used to validate the WHODAS 2.0 (ie, the HAI, PSS, MADRS-S, GAD-7, SDS, and WAI) have, at least in conventional form, been shown to be associated with non-self-reported validators such as clinical diagnoses, sick leave, and health care consumption (eg, [16,37-41]).

We also had no control over what equipment the patients used to access the Web-based WHODAS 2.0, meaning we could not determine the significance of filling in the questionnaire via a mobile phone app rather than a conventional browser on a desktop computer, for example. The results showing good psychometric properties of the WHODAS 2.0 also suggest that there was no substantial measurement error related to the type of device used. Another threat to the generalizability of our findings is that the two samples were relatively homogenous due to the eligibility criteria for the two clinical trials, which were not primarily designed to study the psychometric properties of the WHODAS 2.0. Therefore, it is preferable to validate the findings of this study, particularly with regard to factor structure, in anxiety and stress disorder samples recruited through other means.

### Comparison With Prior Work

The item mean score profile seen in this study—with substantial functional impairment in the “understanding and communicating,” “life activities,” and “getting along with others” domains but very low functional impairment in the “self-care” domain—is highly similar to the item mean score profile seen in studies of interview and pencil-and-paper versions of the WHODAS 2.0 administered to individuals with common mental disorders [8,14]. This suggests that the response pattern does not change much due to the online format.

Before this study, the 12-item WHODAS 2.0 had also been administered online to individuals with common anxiety, stress, and mood disorders in several clinical trials (Table 7, see Multimedia Appendices 1 and 2 for details).

**Table 7:** Previous studies that have administered the 12-item self-report WHODAS 2.0 online to respondents with common mental disorders^a^.

Author (year)	N	Diagnosis^b^	Mean			Cronbach alpha	WHODAS 2.0^c^ change over time?
			12-60 scale^d^	0-48 scale^d^	0-100 scale^d^		
Allen et al (2016) [42]	63	PD	26.0	14.0	29.2	.89	Yes
Andrews et al (2011) [43]	37	SAD	26.6	14.6	30.4	—	Yes
Mason & Andrews (2014) [44]	173^e^	Mixed CMD	25.0	13.0	27.1	—	Yes
Mewton et al (2012) [45]	588	GAD	25.7	13.7	28.5	.90	Yes
Newby et al (2016a) [46]	2109	Mixed CMD	26.3	14.3	29.8	>.88	Yes
Newby et al (2016b) [47]	16	SHA	20.3	8.3	17.3	.83	Yes
Perini et al (2008) [48]	13	MDD	—^f^	—^f^	—^f^	—	Yes
Spence et al (2011) [49]	244	PTSD	32.1	20.1	41.9	.92	—
Titov et al (2008a) [50]	105	SAD	26.3	14.3	29.8	—	Yes
Titov et al (2008b) [51]	88	SAD	25.0	13.0	27.1	—	Yes
Williams et al (2013) [52]	69	MDD	41.2	29.2	60.8	—	Yes
Williams et al (2014) [53]	560	SAD	39.3	27.3	56.9	—	Yes
Williams et al (2015) [54]	75	MDD	44.8	32.8	68.3	.83	Yes
Wootton et al (2011) [55]	118	OCD	30.4	18.4	38.3	.91	—

^a^Information from articles complemented by personal communication via email.

^b^PD: panic disorder; SAD: social anxiety disorder; CMD: common mental disorder; GAD: generalized anxiety disorder; SHA: severe health anxiety; MDD: major depressive disorder; PTSD: posttraumatic stress disorder; OCD: obsessive compulsive disorder; . See Multimedia Appendices 1 and 2 for details.

^c^WHODAS 2.0: World Health Organization Disability Assessment Schedule 2.0.

^d^The 12-60 scale has items scored 1-5, the 0-48 scale has items scored 0-4, and the 0-100 scale is the 0-48 scale divided by 48 and then multiplied by 100.

^e^Another sample (n=135) in this study completed the WHODAS 2.0 on a computer, but not via the Internet.

^f^Unknown; author could not be reached.

Although the primary aim of these trials was not to study the psychometric properties of the WHODAS 2.0, these studies presented estimates of both internal consistency (Cronbach alpha≥.83) and baseline mean scores that are very much in line with data in this trial, and thus lend further support to the validity and generalizability of our findings.

### Conclusions

This is, to date, the most extensive investigation into the psychometric properties of the self-rated 12-item version of the WHODAS 2.0 when administered via the Internet to individuals with anxiety and stress disorders. When administered online to individuals with anxiety and stress disorders, the WHODAS 2.0 exhibits high internal consistency, high convergent validity, adequate test-retest reliability, and is sensitive to change. We conclude that the psychometric properties of the self-rated 12-item version of the WHODAS 2.0 are acceptable when the instrument is administered via the Internet to individuals with anxiety and stress disorders, but suggest that the three subfactors found in this study be reported alongside the sum score to facilitate comparisons between studies.

## Appendix

Multimedia Appendix 1 Search strategy and data collection for brief review.

Multimedia Appendix 2 Flowchart illustrating study selection process for brief review.

## Abbreviations

AUC: area under the curve
CBT: cognitive behavior therapy
CFI: comparative fit index
DSM-5: Diagnostic and Statistical Manual of Mental Disorders (Fifth Edition)
GAD-7: Generalized Anxiety Disorder seven-item scale
HAI: Health Anxiety Inventory
ICC: intraclass correlation coefficient
ICD-10: International Classification of Diseases, Tenth Revision
ITC: item-total correlations
MADRS-S: Montgomery-Åsberg Depression Rating Scale-self-report
PSS: Perceived Stress Scale
RMSEA: root mean square error of approximation
SDS: Sheehan Disability Scale
SRMR: standardized root mean square residual
TLI: Tucker-Lewis index
WAI: Work Ability Index
WHODAS 2.0: World Health Organization Disability Assessment Schedule 2.0
