# Supplementary material for: The 12-item Self-Report World Health Organization Disability Assessment Schedule (WHODAS) 2.0 Administered Via the Internet to Individuals With Anxiety and Stress Disorders: A Psychometric Investigation Based on Data From Two Clinical Trials
Source: JMIR Ment Health. 2017 Dec 8;4(4):e58. doi: 10.2196/mental.7497 (PMC5741825; doi:10.2196/mental.7497)
Supplement: Multimedia Appendix 1 [file mental_v4i4e58_app1.pdf]

Multimedia Appendix 1

As previously published evidence pertaining to the reliability and validity of using the web-based self-report WHODAS 2.0 to assess individuals with anxiety-, stress-, and mood disorders is scattered and fragmentary, we assembled published data for a brief systematic review (Table 7 in the main article). We did not expect published studies to include factor analyses or estimates of test-retest reliability, but focused on finding results related to internal consistency and as well as within-group change. A secondary aim of the review was to tabulate published online WHODAS 2.0 mean scores seen in common psychiatric disorders, so as to facilitate future comparisons between studies, especially in the light of conflicting scoring algorithms.

We searched PubMed, PsycInfo, and Web of Science for published English-language scientific papers presenting data where the 12-item self-report WHODAS 2.0 had been administered online to individuals with common mental disorders. Search strings (see below) used the "AND" operator to combine terms related to the WHODAS 2.0 with terms related to information technology (e.g., "online"). No filters or time restrictions were imposed. As we found no medical subject headings or psychological index terms of relevance, we focused on searching through titles, abstracts, and keywords. We also searched for terms related to the WHODAS 2.0 in all issues of Internet Interventions, a journal specialized in research pertaining to the application of internet-based technology in mental- and behavioral health. All sources were searched for the last time on November 28th, 2016.

PubMed

(WHODAS*[All Fields] OR ”WHO Disability Assessment Schedule"[All Fields] OR ”World Health Organization Disability Assessment Schedule"[All Fields]) AND (internet[All Fields] OR online[All Fields] OR web[All Fields])

PsycInfo

("WHODAS*".mp. or "WHO Disability Assessment Schedule".mp. or "World Health Organization Disability Assessment Schedule".mp.) and (exp internet/ or exp online therapy/ or exp online experiments/ or "internet".mp. or "online".mp. or "web".mp.)

Web of Science

TOPIC: (WHODAS* OR "WHO Disability Assessment Schedule" OR "World Health Organization Disability Assessment Schedule") AND (internet OR online OR web)

Internet Interventions

“WHODAS” / "WHO Disability Assessment Schedule" / "World Health Organization Disability Assessment Schedule"

The first author read all papers in full-text to determine if they were suitable for the brief review. Eligibility criteria were: (*a*) using the 12-item self-report WHODAS 2.0, (*b*) web-based administration, (*c*) adult respondents with common mental disorders, (*d*) results with relevance to the reliability, validity and norms of the WHODAS 2.0, and (*e*) no other primary paper reporting the same relevant data. In those cases where information was missing or difficult to extract, authors were contacted via e-mail for additional information. In the case of double reporting or overlapping samples in two or more articles an attempt was made to exclude those papers that were deemed least comprehensive with regard to reporting of relevant data. We did not impose any further restrictions with regard to study design or type. The paper inclusion process is illustrated in the form of a flowchart (Multimedia Appendix 2).
