# Supplementary figures and images for: The 12-item Self-Report World Health Organization Disability Assessment Schedule (WHODAS) 2.0 Administered Via the Internet to Individuals With Anxiety and Stress Disorders: A Psychometric Investigation Based on Data From Two Clinical Trials
Source: JMIR Ment Health. 2017 Dec 8;4(4):e58. doi: 10.2196/mental.7497 (PMC5741825; doi:10.2196/mental.7497)

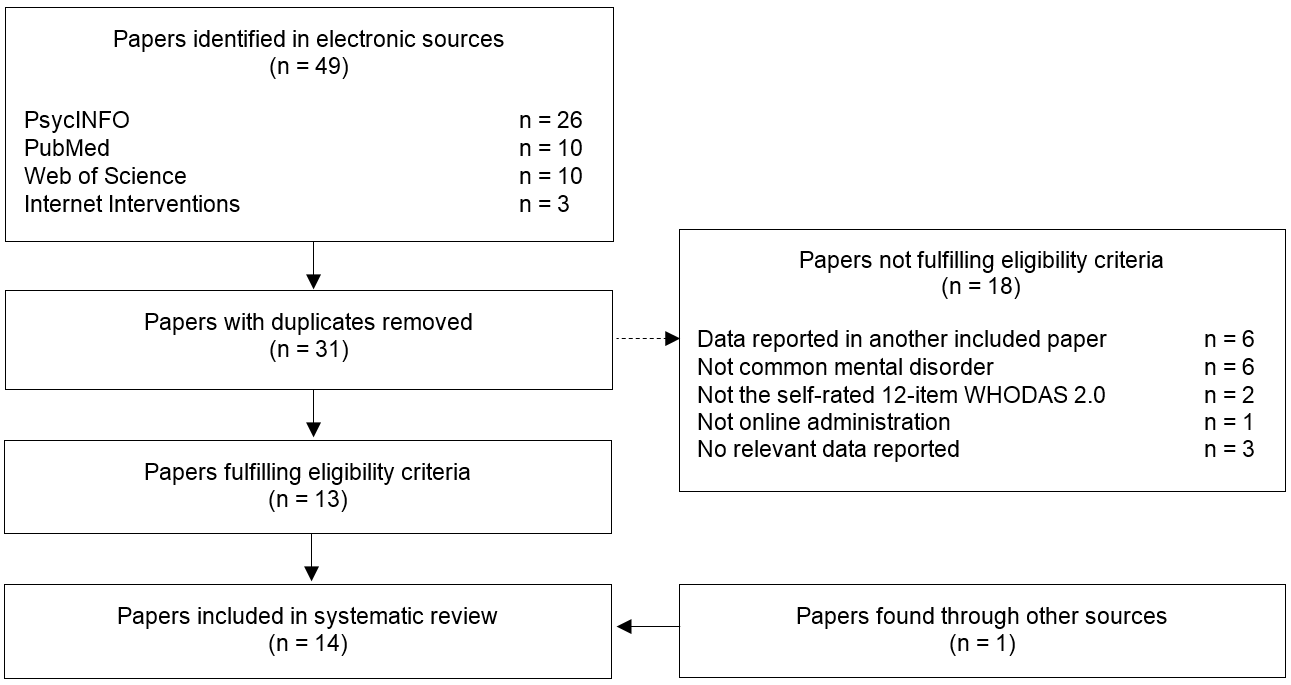

Supplement: Multimedia Appendix 2 [file mental_v4i4e58_app2.png]
